# Effects of Mycorrhizal Colonization on Transcriptional Expression of the Responsive Factor *JERF3* and Stress-Responsive Genes in Banana Plantlets in Response to Combined Biotic and Abiotic Stresses

**DOI:** 10.3389/fpls.2021.742628

**Published:** 2021-10-28

**Authors:** Younes M. Rashad, Waleed M. E. Fekry, Mohamed M. Sleem, Nahla T. Elazab

**Affiliations:** ^1^Plant Protection and Biomolecular Diagnosis Department, Arid Lands Cultivation Research Institute, City of Scientific Research and Technological Applications (SRTA-City), New Borg El-Arab City, Egypt; ^2^Plant Production Department, Arid Lands Cultivation Research Institute, City of Scientific Research and Technological Applications (SRTA-City), Alexandria, Egypt; ^3^Botany Department, Faculty of Science, Mansoura University, Mansoura, Egypt

**Keywords:** arbuscular mycorrhiza, banana, *Fusarium solani*, plant resistance, salinity

## Abstract

Banana plants (*Musa acuminata* L.) are exposed to various biotic and abiotic stresses that affect their production worldwide. Banana plants respond to these stresses, but their responses to combined stresses are unique and differ from those to various individual stresses. This study reported the effects of the mycorrhizal colonization of banana roots and/or infection with root rot on the transcriptional expression of the responsive factor *JERF3* and stress-responsive genes (*POD, PR1*, *CHI*, and *GLU*) under different salinity levels. Different transcriptional levels were recorded in response to the individual, dual, or triple treatments. All the applied biotic and abiotic stresses triggered the transcriptional expression of the tested genes when individually applied, but they showed different influences varying from synergistic to antagonistic when applied in combinations. The salinity stress had the strongest effect when applied in combination with the biotic stress and/or mycorrhizal colonization, especially at high concentrations. Moreover, the salinity level differentially affects the banana responses under combined stresses and/or mycorrhizal colonization in addition, the mycorrhizal colonization of banana plantlets improved their growth, photosynthesis, and nutrient uptake, as well as greatly alleviated the detrimental effects of salt and infection stresses. In general, the obtained results indicated that the responses of banana plantlets under the combined stresses are more complicated and differed from those under the individual stresses depending on the crosstalks between the signaling pathways.

## Introduction

Banana (*Musa acuminata* L.) is one of the most important tropical fruit crops worldwide and a major economic commodity for some countries in international trading. In 2019, the global export of bananas was around 24.7 million tons with a total value of USD 13 billion, representing the highest export value among the international trade of fruits. In Egypt, 1.36 million tons of bananas were produced in 2019 for a production area of around 30.4 ha (FAOSTAT, 2021). Moreover, bananas have a high nutritional content of carbohydrates, proteins, potassium (K), calcium, phosphorus (P), nitrogen (N), and vitamins, in addition to their medicinal importance (Ranjha et al., 2020). However, bananas are exposed to diverse biotic and abiotic stresses which affect their production.

Banana root rot, caused by *Fusarium solani* (Mart.) Sacc., is one of the soil-borne diseases that affect crop yield. The disease symptoms include rotting and lesions in the root system that result in reducing the water and nutrient uptake and decreasing plant growth and productivity (El-Nagdi et al., 2015). Various studies were conducted to control banana root rot using chemical fungicides such as benomyl, carbendazim, mancozeb, and prochloraz, and biocontrol agents such as *Streptomyces* spp., *Bacillus* spp., *Pseudomonas* spp., *Trichoderma* spp., and *Saccharomyces* spp. (El-Deeb and El-Naggar, 2008).

Salinity is considered one of the main abiotic stresses threatening global agriculture including bananas, being a limiting factor for plant growth and productivity. The adverse effects of salinity on plant physiology and biochemistry include osmotic stress. This leads to water stress, and ion cytotoxicity stress caused by the high sodium (Na) ion uptake. The high uptake of sodium ions results in a decrease in the uptake of K and calcium ions causing the inhibition of the enzymatic activities and the cellular functions (Isayenkov and Maathuis, 2019). In addition, oxidative stress can also occur due to the production of reactive oxygen species (ROS) that attack plant tissues and DNA (Kamran et al., 2020). In contrast, plants have several defense mechanisms in response to these biotic and abiotic stresses such as the activation of specific ion channels and kinase cascades, and signaling phytohormones such as abscisic acid (ABA), jasmonic acid (JA), salicylic acid (SA), and ethylene (ET) but their efficiency and diversity differ according to the plant susceptibility to those stresses (Ben Rejeb et al., 2014). However, plant responses to combined stress are unique and may be different from their response to individual stresses. In other words, the combined stresses lead to high levels of complexity in the plant responses, which are mediated by different synergistic and/or antagonistic signaling pathways (Suzuki et al., 2014).

The ethylene response factor *JERF3*, which belongs to the *ERF* plant-specific transcription factor family, has significant roles in regulating multiple responsive genes against different biotic and abiotic stresses, as well as plant growth and development genes *via* the JA and ET signaling pathways (Pegoraro et al., 2013). Furthermore, different defensive genes are overexpressed in plants in response to various biotic and abiotic stresses such as the peroxidase gene (*POD*). The *POD* is involved in many developmental and defensive processes and has antioxidant activities by oxidizing the phenolic compounds regulating ROS and the free radicals produced by abiotic and biotic stresses (Shigeto and Tsutsumi, 2016). The antifungal gene *PR1* encodes a protein with antifungal activity and is involved in plant resistance against many fungal pathogens (Breen et al., 2017). The Chitinase gene (*CHI*) encodes the chitinase enzyme that is involved in the plant defense against different biotic and abiotic stresses via the JA-signaling pathway (Zheng et al., 2020). The Glucanase gene (*GLU*) encodes the antifungal enzyme *β*-1,3-glucanase and is also involved in the plant resistance against different stresses *via* the SA-signaling pathway (Gupta et al., 2013).

Arbuscular mycorrhizal fungi are obligate symbionts, which form mutualistic associations with roots of the majority of terrestrial plants. Various functional roles have been reported for arbuscular mycorrhizal fungi (AMF) colonization of the host plant including growth promotion (Rashad et al., 2020b), enhancement of nutrient uptake (El-Sharkawy et al., 2018), improvement of plant tolerance to abiotic stresses such as salinity and drought (Evelin et al., 2019), and induction of plant defense-responses against different pathogens (Aseel et al., 2019; Rashad et al., 2020a). In this regard, Yano-Melo et al. (2003) found that the colonization of banana plants with AMF, especially *Glomus clarum*, significantly reduced the salt inhibitory effect on the plant growth parameters more effectively if the salinity is ≤4.68 dS/m, compared with the non-mycorrhizal plants. Moreover, SuChen et al. (2012) reported a considerable reduction of up to 67% in the incidence of *Fusarium* wilt disease, caused by *F. oxysporum* f. sp. *cubense*, in banana plants colonized with *G. clarum*. However, limited studies have investigated the effects of mycorrhizal colonization on plant responses to combined biotic and abiotic stresses. The present study aimed to investigate effects of mycorrhizal colonization on transcriptional expression of the responsive factor *JERF3* and stress-responsive genes *POD*, *PR1*, *GLU*, and *CHI* in banana plantlets in response to combined and individual stresses of salinity and root rot infection. In addition, effects on the growth, biochemical parameters, and disease development of the stressed banana plantlets were studied.

## Materials and Methods

### Banana Cultivar and Fungal Inocula

Tissue-cultured banana plantlets (*M. acuminata* cv. Grand Nain) were obtained from the Genetic Engineering and Biotechnology Research Institute, University of Sadat City, Egypt. An isolate of the pathogenic fungus *F. solani*, isolated from banana roots exhibiting root rot symptoms, was used in the greenhouse experiment. For the inoculum preparation, the pathogenic fungus was grown in glass flasks containing a mixture of sterilized sand and ground maize grains (1:2 v/v) and incubated at 28°C for 2 weeks.

For the AMF inoculum preparation, a mixture of AMF including *Rhizoglomus clarum* (T.H. Nicolson and N.C. Schenck) Sieverd. G.A. Silva and Oehl, *Funneliformis mosseae* (T.H. Nicolson & Gerd.) C. Walker and A. Schüßler, and *Rhizophagus aggregatus* (N.C. Schenck & G.S. Sm.) C. Walker, in the equal ratio, was used. The AMF inoculum, which consisted of colonized root pieces and rhizospheric soil containing AMF spores and external mycelia, was propagated in pot cultures using sudangrass, at a 75% colonization index. The cultures were grown for two cycles (each of 4 months) at 25 ± 2°C, 65% humidity, and in a 12 h day /12 h night photoperiod. The sudangrass plants were then harvested and the rhizospheric soil and the colonized roots were used as the AMF inoculum.

### Greenhouse Experiment

Pots (30 cm diameter) filled with sterilized soil (sand mixed with clay at a ratio of 1:2 v/v) were used in this experiment. Forty-five-day-old banana plantlets were transplanted in the prepared pots at five plantlets per pot. Two weeks after transplanting, soil infection was done by mixing the pathogen inoculum with the soil upper layer of the pots at 3% (v/v). Half of them were inoculated twice with AMF at the transplanting time (10 g per plantlet) and with a soil drench 30 days after transplantation (15 ml per plantlet) using AMF spore suspension (1 × 10^6^ unit L^–1^). Solutions with three levels of salinity (0.7, 2.3, and 3.5 dS m^–1^) were applied. Ten pots were used as replicates for each treatment. All the pots were regularly irrigated as required, arranged in a factorial split–split plot design (2 × 3 × 2), i.e., two treatments of mycorrhization (+AM, and −AM), three levels of salinity (S0 = 0.7, S1 = 2.3, and S2 = 3.5 dS m^–1^), and two treatments of infection (+ P, and −P), and kept in the greenhouse at 28/20°C day/night temperature at 70% humidity.

### Quantitative Real-Time PCR (qPCR)

Two weeks after the pathogen inoculation (api), three banana plantlets were uprooted, washed under running tap water to remove the soil particles, and the total RNA was extracted from the root system using an RNeasy Mini Kit (Qiagen, Germany) according to the instructions of the manufacturer. The extracted RNA was incubated with DNase for 1 h at 37°C.

For the complementary DNA (cDNA) synthesis, a reverse transcription (RT) -PCR kit (Qiagen, Germany) was used according to the instructions of the manufacturer. The reaction mixture (20 μl) contained 2.5 μl of dNTPs (2.5 mM), 5 μl of 5X-buffer with MgCl_2_, 4 μl of oligo (dT) primer (20 pmol. μl^–1^), 0.2 μl of reverse transcriptase enzyme (Omniscript RT, Qiagen, Germany), and 2 μl of RNA. The PCR amplification was performed using a thermal cycler (Promega, Germany), at 42°C for 2 h and 65°C for 20 min.

The qPCR mixture (20 μl) included 1 μl of the template, 12.5 μl of SYBR Green Master Mix (Bioline, Germany), 1 μl of forward primer, 1 μl of reverse primer, and sterile RNase free water. *β*-actin was used as a reference gene (*β*-actin-F 5′-GTGGGCCGCTCTAGGCACCAA-3′, and *β*-actin-R 5′-CTCTTTGATGTCACGCACGATTTC-3′). Five pairs of primers, selected as pathway-reporter genes, were used in this study and are presented in Table 1. The reaction was performed using a Rotor-Gene-6000-system (Qiagen, United States) as follows: one cycle at 95°C for 10 min, 40 cycles (95°C for 20 s, 58°C for 25 s, and 72°C for 30 s). For each sample, three biological and three technical replicates were performed. The comparative CT method (2^–ΔΔCT^) was used to analyze the relative mRNA expression levels according to Livak and Schmittgen (2001).

**TABLE 1:** Primer sequences of the five genes used in the quantitative real-time PCR (qPCR).

Gene description	Abbrev.	Accession No.	Sequence (5′-3′)
Jasmonate and ethylene-responsive factor 3	*JERF3*-F	AY383630	GCCATTTGCCTT CTCTGCTTC
	*JERF3*-R		GCAGCAGCATC CTTGTCTGA
Pathogenesis-related-protein 1	*PR1*-F	M69247	ACTTGGCATCCCG AGCACAA
	*PR1*-R		CTCGGACACCC ACAATTGCA
Chitinase class II	*PR3*-F	U30465	GCGTTGTGGTTCT GGATGACA
	*PR3*-R		CAGCGGCAGAA TCAGCAACA
*β*-1,3-glucanase	*PR2*-F	M80604	TTTCGATGCCCT TGTGGATT
	*PR2*-R		CGGCCAACCACT TTCCGATAC
Peroxidase	*POD*-F	X94943	CCTTGTTGGTGGG CACACAA
	*POD*-R		GGCCACCAGTG GAGTTGAAA

### Plant Growth Evaluation

Four and 10 weeks api, three banana plantlets from each treatment were carefully uprooted, washed with tap water, and evaluated for the shoot and root lengths, shoot and root dry weights, and leaf area. The plant samples were oven-dried at 80°C for 48 h before the constant dry weights were recorded.

### Estimation of Nutrients Content

The total N content in the banana leaves was estimated using the modified Kjeldahl method (Peach and Tracy, 1955). The total P content was determined by the spectrophotometric vanadium phosphomolybdate method (Wild, 1988) using a spectrophotometer (Unico 2150-UV, United States). The K and Na estimation (Cottenie et al., 1982) was carried out using a flame photometer (SLE-S-935, India), while chloride (Cl) content was estimated using the potentiometric titration method (Jackson, 2005).

### Estimation of Photosynthetic Pigments

Four and 10 weeks api, the photosynthetic pigments (chlorophyll *a*, chlorophyll *b*, and carotenoids) were estimated in banana leaves using the spectrophotometric method as described by Harborne (1984).

### Disease Assessment

All the banana plantlets were assessed for root rot disease incidence (DI) and severity (DS) 4 and 10 weeks api. The DS was estimated by rating the root system (primary and lateral roots) rotting and necrosis areas of each banana plantlet according to the modified 6-point grading scale of Carling et al. (1999), where 0 = no damage and 5 = full death using the following equation:


$$\text{DS}\left(\%\right)=\frac{\Sigma \,\left(a\,b\right)}{A\,K}\times 100$$

Where, *a* = number of plantlets with the same infection degree, *b* = infection degree, *A* = total number of the evaluated banana plantlets, and *K* = highest infection degree. DI was estimated according to the following equation:


$$\text{DI}\;(\%)=\frac{\mathrm{Number}\,\mathrm{of}\,\mathrm{infected}\,\mathrm{plantlets}}{\mathrm{Total}\,\mathrm{number}\,\mathrm{of}\,\mathrm{plantlets}}\times 100$$

### Estimation of Mycorrhizal Colonization

For each treatment, five banana plantlets were carefully uprooted and washed with tap water, 4 and 10 weeks api to estimate the mycorrhizal colonization. The root system was cut into small pieces (1 cm) and stained with 0.05% trypan blue (Sigma, St. Louis, MO, United States) according to Phillips and Hayman (1970). The mycorrhizal colonization was estimated according to Trouvelot et al. (1986). For each treatment, forty stained segments of banana roots were mounted on glass slides in lactoglycerol and examined using a light microscope (at × 100 and × 400 magnifications) for the estimation of the frequency of root colonization (F, %), the intensity of cortical colonization (I, %), and the frequency of arbuscules (A, %).

### Statistical Analyses

The data were statistically analyzed using the software CoStat (v 6.4). Comparisons between the means were made using Duncan’s multiple range test at *p* ≤ 0.05 (Duncan, 1955).

## Results

### Transcript Levels of Some Stress-Responsive Genes in Response to Different Stresses

**FIGURE 1:**
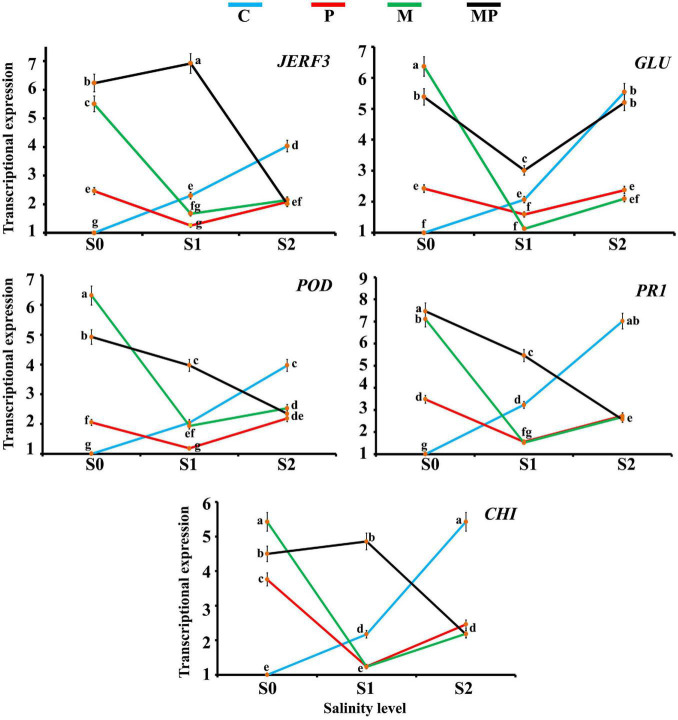
Line charts exhibiting the transcriptional expression levels of five defense-related genes in banana roots in response to mycorrhizal colonization and/or infection with root rot under different levels of salinity. Where, C = non-mycorrhizal-uninfected plantlets, P = non-mycorrhizal-infected plantlets, M = mycorrhizal-uninfected plantlets, and MP = mycorrhizal-infected plantlets. In each subfigure, the treatments marked with the same letter are not significantly different at *p* ≤ 0.05 according to Duncan’s multiple range test (Duncan, 1955). The bars represent the SE. Each value represents the mean of three biological replicates, each analyzed in triplicate.

The transcriptional expressions of the responsive factor *JERF3* and four stress-responsive genes in banana roots in response to colonization with AMF and/or infection with root rot under different levels of salinity were investigated (Figure 1). For *JERF3*, the data obtained from the qPCR indicated that the gene expression was directly proportional to the salinity level exhibiting the inducing effect of the salinity level on the gene expression. The infection with root rot also triggered the gene expression under the salinity levels S0 and S2, but not under the salinity level S1, where the gene expression was extremely down-regulated. However, the inducing effect of the salinity stress was higher than that of the fungal infection. The colonization with AMF led to a considerable induction in the gene expression under the salinity level S0 (5.5-fold). The gene expression in the mycorrhizal banana roots under the salinity level S1 was lower than in that under S0 and S2 levels. At the same time, the gene expression was highly induced in the mycorrhizal-infected banana plantlets under the salinity level S0 (6.2-fold). The maximum gene expression was recorded in the mycorrhizal-infected banana plantlets under the salinity level S1 (6.9-fold), but the inducing effect was lower in the mycorrhizal banana plantlets infected with root rot under salinity level S2 than the other salinity levels.

For *GLU*, the obtained results indicated that the salinity level directly triggered the gene expression with the maximum triggering effect at salinity level S2 (5.5-fold). The infection with root rot induced the gene expression under different salinity levels but the gene expression was lower under salinity level S1 than under salinity levels S0 and S2. The mycorrhizal colonization led to a high induction in the gene expression under salinity levels S0 and S2, recording the highest gene expression level (6.4-fold) under salinity level S0. Downregulation of the gene expression was observed in the mycorrhizal roots under salinity level S1. The mycorrhizal colonization of the infected banana roots upregulated the gene expression under all the tested salinity levels, compared with the non-mycorrhizal treatments, but the expression level was lower under salinity level S1 than S0 and S2.

For *POD*, the gene expression was upregulated with the increase in the salinity level. The infection with root rot also upregulated the gene expression level under the salinity levels S0 and S2 but downregulated under salinity level S1. The mycorrhizal colonization of banana roots under salinity level S0 highly induced the gene expression, recording the highest gene expression (6.3-fold). The inducing effect was lower in the mycorrhizal roots under salinity level S1 than that under salinity level S2. The mycorrhizal colonization of the infected roots upregulated the gene expression, but the expression level was inversely proportional with the salinity level.

For *PR1*, the gene expression was directly proportional to the salinity level. In this regard, the highest expression was recorded under salinity level S2. The infection with root rot also induced the gene expression under salinity levels S0 and S2, but the gene expression declined under salinity level S1. The mycorrhizal colonization of the banana roots upregulated the gene expression under salinity levels S0 and S2, but not under salinity level S1. The mycorrhizal colonization of the infected roots highly upregulated the gene expression, recording the highest expression level under salinity level S0 (7.5-fold), but this inducing effect lowered with the increase in the salinity level.

For *CHI*, the gene expression was directly proportional to the salinity level. In this regard, the highest expression level was recorded under salinity level S2 (5.4-fold). The infection of the banana roots with root rot upregulated the gene expression under salinity levels S0 and S2, but not under salinity level S1. The mycorrhizal colonization of the banana roots triggered the gene expression under salinity level S0, recording the highest expression level (5.4-fold), higher than under salinity level S2, but not under salinity level S1. The mycorrhizal colonization of the infected roots induced the gene expression under different salinity levels, but the expression level was higher under salinity level S1 than S0 and both of them were higher than that under salinity level S2.

### Effects on the Plant Growth

**FIGURE 2:**
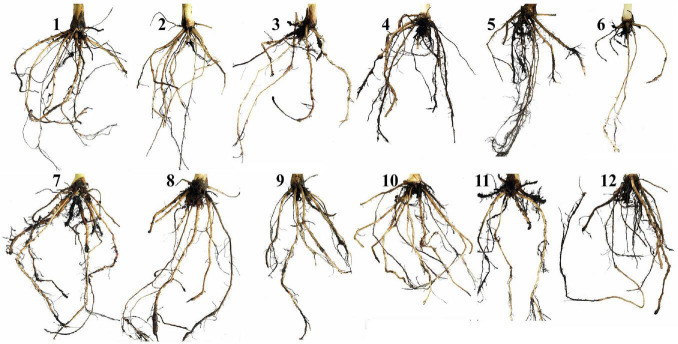
A photograph showing the impacts of the different individual and combined stresses on the morphology of banana root system. Where, 1 = untreated control plantlets under salinity level S0, 2 = plantlets under salinity level S1, 3 = plantlets under salinity level S2, 4 = infected plantlets with root rot under salinity level S0, 5 = infected plantlets with root rot under salinity level S1, 6 = infected plantlets with root rot under salinity level S2, 7 = mycorrhizal plantlets under salinity level S0, 8 = mycorrhizal plantlets under salinity level S1, 9 = mycorrhizal plantlets under salinity level S2, 10 = mycorrhizal-infected plantlets under salinity level S0, 11 = mycorrhizal-infected plantlets under salinity level S1, and 12 = mycorrhizal-infected plantlets under salinity level S2.

Means of the evaluated growth parameters of banana plantlets in response to the colonization with AMF and/or infection with root rot under different levels of salinity are presented in Table 2. The data obtained from the greenhouse experiment showed that salinity level reduced all the evaluated growth parameters including the shoot and root system lengths and dry weights. The leaf area of banana plantlets was also reduced. The rate of reduction in these parameters was directly proportional to the salinity level. Although the reduction due to salinity continued from 4 to 10 weeks api, the reduction was more effective with the increase in the plant age. The infection of banana plantlets with root rot negatively affected plant growth. The negative effects due to the infection were more evident at 10 weeks than at 4 weeks api. However, the colonization of banana roots with AMF remarkably enhanced all growth parameters achieving the highest values of these parameters compared with the non-mycorrhizal plantlets, whether at 4 or 10 weeks api. The mycorrhizal banana plantlets exhibited thicker and more vigorous roots with heavier lateral growth than the non-mycorrhizal ones. Moreover, the colonization with AMF significantly alleviated the negative effects due to the salinity stress and/or infection with root rot, when compared with the non-mycorrhizal banana plantlets subjected to any or both of these stresses. However, the mitigation effect of the AMF colonization lowered with the increase in the salinity level and was higher in the individual stresses than the combined stresses. The root system was more affected by the tested treatments than the shoot system, in particular the morphological characteristics. Varied impacts of the different individual and combined stresses on the morphology of banana root system are illustrated in Figure 2. The salinity level or infection with root rot negatively affected the thickness, density, and lateral growth of the banana roots. The root colonization with AMF enhanced these morphological characteristics in the unstressed banana plantlets and mitigated the negative effects in case of the stressed ones. However, the morphological effects due to the combined stresses were more severe than due to the individual stresses with the root system becoming thinner, darker in color, and longer than the banana roots under the combined stresses than under the individual stresses.

**TABLE 2:** Growth parameters of banana plants in response to colonization with arbuscular mycorrhizal fungi and/or infection with *Fusarium* root rot under different levels of salinity^*a*^.

Weeks after pathogen inoculation	Mycorrhizal status	Salinity level	Infection	Shoot length (cm)	Root length (cm)	Shoot dry wt. (g)	Root dry wt. (g)	Leaf area (cm^2^)
4	−M	S0	−P	23.7 ± 3.51	40.3 ± 2.53	1.84 ± 0.36	0.43 ± 0.01	95.1 ± 1.1
			+P	19.7 ± 1.53	33.3 ± 2.23	1.54 ± 0.23	0.40 ± 0.09	91.3 ± 0.8
		S1	−P	21.7 ± 0.97	34.0 ± 3.10	1.65 ± 0.23	0.33 ± 0.07	95.1 ± 1.0
			+P	17.0 ± 1.10	26.3 ± 1.57	0.95 ± 0.06	0.31 ± 0.08	89.3 ± 0.3
		S2	−P	17.0 ± 1.30	23.0 ± 1.80	1.56 ± 0.25	0.24 ± 0.06	77.2 ± 1.4
			+P	13.7 ± 1.53	18.1 ± 2.58	0.91 ± 0.08	0.24 ± 0.05	71.2 ± 1.0
	+M	S0	−P	29.0 ± 2.30	45.7 ± 2.51	2.62 ± 0.37	0.56 ± 0.09	100.9 ± 1.3
			+P	21.7 ± 2.10	40.5 ± 2.30	1.58 ± 0.25	0.47 ± 0.08	94.6 ± 0.8
		S1	−P	23.3 ± 3.40	40.3 ± 3.12	1.67 ± 0.21	0.44 ± 0.08	99.3 ± 0.3
			+P	20.7 ± 1.63	30.1 ± 1.51	1.30 ± 0.31	0.38 ± 0.07	95.0 ± 0.3
		S2	−P	19.0 ± 2.70	25.0 ± 2.31	1.50 ± 0.22	0.35 ± 0.02	88.8 ± 1.4
			+P	15.0 ± 1.50	20.3 ± 1.56	1.22 ± 0.30	0.32 ± 0.04	82.9 ± 0.9
10	−M	S0	−P	32.0 ± 2.07	51.7 ± 3.16	4.01 ± 0.71	0.90 ± 0.05	103.2 ± 0.9
			+P	25.3 ± 2.81	40.3 ± 2.78	3.20 ± 0.63	0.82 ± 0.06	96.4 ± 0.7
		S1	−P	24.2 ± 1.68	40.7 ± 2.50	3.77 ± 0.57	0.75 ± 0.06	100.7 ± 1.0
			+P	21.1 ± 2.01	31.7 ± 1.98	2.52 ± 0.26	0.71 ± 0.02	98.4 ± 1.1
		S2	−P	21.5 ± 1.95	31.8 ± 2.04	3.03 ± 0.55	0.69 ± 0.07	94.5 ± 1.3
			+P	18.7 ± 1.14	25.5 ± 2.63	2.47 ± 0.21	0.65 ± 0.07	85.1 ± 0.9
	+M	S0	−P	38.6 ± 2.32	60.1 ± 3.44	4.58 ± 1.00	1.12 ± 0.11	114.9 ± 1.8
			+P	29.7 ± 1.98	49.3 ± 3.52	3.41 ± 0.81	0.91 ± 0.08	103.4 ± 0.9
		S1	−P	27.2 ± 1.84	47.7 ± 2.74	3.97 ± 0.80	0.87 ± 0.06	104.8 ± 0.7
			+P	23.3 ± 1.79	37.6 ± 2.35	3.07 ± 0.77	0.81 ± 0.05	102.3 ± 0.9
		S2	−P	23.7 ± 1.53	34.2 ± 2.19	3.14 ± 0.63	0.79 ± 0.07	98.3 ± 0.8
			+P	19.6 ± 1.51	27.1 ± 1.99	2.99 ± 0.58	0.70 ± 0.05	90.6 ± 0.6
LSD 0.05				3.68	4.99	0.29	0.07	3.37
Mycorrhiza				*	*	*	*	*
Salinity				***	***	***	***	**
Mycorrhiza × Salinity				*	**	*	**	**
Infection				**	**	**	**	**
Infection × Mycorrhiza				*	*	*	**	**
Infection × Salinity				**	**	**	***	*
Infection × Salinity × Mycorrhiza				**	**	**	***	**

*^a^Values are the means of three replicates ± SD, where, −AM = non-mycorrhizal, +AM = mycorrhizal, S_0_ = salinity level of 0.7, S_1_ = salinity level of 2.3, S_2_ = salinity level of 3.5 dS m^–1^, −P = non-infected, and +P = infected with *Fusarium* root rot.*

* = significant at p < 0.05, ** = significant at p < 0.01, and *** = significant at p < 0.001.

### Effects on the Nutrient Content

The mineral nutrient contents in the shoots of banana plantlets in response to the colonization with AMF and/or infection with root rot under different levels of salinity are presented in Table 3. The results from the greenhouse experiment showed that the N, P, and K contents declined in the banana leaves with the increase in the salinity level more after 10 than 4 weeks api, compared with the untreated control plantlets. The infection with root rot led to a reduction in these nutrient contents when compared with the uninfected banana plantlets. The Na and Cl contents increased in the banana leaves with the increase in the salinity level and/or due to the infection with root rot. The colonization with AMF increased the banana leaves contents of these two nutrients as well compared with the non-mycorrhizal plantlets, with a higher content after 10 than 4 weeks api. At the same time, the colonization with AMF mitigated the reduction in the N, P, and K contents due to the infection or salinity, and minimized the elevated Na and Cl contents in the banana leaves compared with the untreated control plantlets.

**TABLE 3:** Mineral nutrient percentages in the shoots of banana plants in response to the colonization with arbuscular mycorrhizal fungi and/or infection with *Fusarium* root rot under different levels of salinity^*a*^.

Weeks after pathogen inoculation	Mycorrhizal status	Salinity level	Infection	Nitrogen (%)	Phosphorus (%)	Potassium (%)	Sodium (%)	Chloride (%)
4	−M	S0	−P	2.55 ± 0.32	0.18 ± 0.05	4.27 ± 0.21	0.014 ± 0.001	0.73 ± 0.02
			+P	2.44 ± 0.41	0.14 ± 0.05	3.76 ± 0.12	0.021 ± 0.009	0.78 ± 0.01
		S1	−P	2.45 ± 0.21	0.14 ± 0.06	3.92 ± 0.11	0.072 ± 0.009	0.81 ± 0.02
			+P	2.34 ± 0.42	0.11 ± 0.04	3.53 ± 0.06	0.081 ± 0.004	0.92 ± 0.02
		S2	−P	2.33 ± 0.12	0.12 ± 0.03	3.35 ± 0.05	0.085 ± 0.06	0.93 ± 0.05
			+P	2.18 ± 0.31	0.10 ± 0.05	3.12 ± 0.08	0.094 ± 0.010	1.17 ± 0.09
	+M	S0	−P	3.58 ± 0.65	0.33 ± 0.04	5.30 ± 0.22	0.015 ± 0.009	0.70 ± 0.03
			+P	3.19 ± 0.28	0.24 ± 0.04	4.57 ± 0.16	0.022 ± 0.007	0.86 ± 0.02
		S1	−P	2.93 ± 0.32	0.18 ± 0.01	3.73 ± 0.08	0.138 ± 0.003	0.92 ± 0.04
			+P	2.81 ± 0.93	0.21 ± 0.02	3.41 ± 0.12	0.019 ± 0.007	0.91 ± 0.01
		S2	−P	2.76 ± 0.32	0.19 ± 0.05	3.13 ± 0.14	0.047 ± 0.005	0.94 ± 0.01
			+P	2.45 ± 0.67	0.15 ± 0.03	2.87 ± 0.09	0.089 ± 0.006	0.97 ± 0.02
10	−M	S0	−P	2.59 ± 0.02	0.19 ± 0.05	4.47 ± 0.06	0.016 ± 0.005	0.75 ± 0.05
			+P	2.52 ± 0.01	0.17 ± 0.05	4.20 ± 0.10	0.028 ± 0.006	0.86 ± 0.01
		S1	−P	2.48 ± 0.01	0.15 ± 0.04	4.13 ± 0.12	0.084 ± 0.008	0.98 ± 0.01
			+P	2.47 ± 0.02	0.16 ± 0.03	3.97 ± 0.12	0.087 ± 0.002	0.79 ± 0.03
		S2	−P	2.39 ± 0.03	0.13 ± 0.01	3.67 ± 0.05	0.088 ± 0.003	1.00 ± 0.05
			+P	2.23 ± 0.02	0.12 ± 0.02	3.41 ± 0.13	0.097 ± 0.002	1.21 ± 0.02
	+M	S0	−P	3.77 ± 0.02	0.35 ± 0.01	5.37 ± 0.18	0.015 ± 0.001	0.70 ± 0.02
			+P	3.54 ± 0.01	0.32 ± 0.03	4.87 ± 0.06	0.024 ± 0.003	0.77 ± 0.05
		S1	−P	3.19 ± 0.02	0.27 ± 0.02	4.18 ± 0.05	0.077 ± 0.003	0.82 ± 0.08
			+P	2.95 ± 0.01	0.23 ± 0.03	4.05 ± 0.10	0.074 ± 0.002	0.87 ± 0.06
		S2	−P	2.84 ± 0.02	0.21 ± 0.02	3.97 ± 0.06	0.081 ± 0.002	0.91 ± 0.08
			+P	2.71 ± 0.01	0.19 ± 0.02	3.63 ± 0.04	0.092 ± 0.006	1.02 ± 0.07
LSD 0.05				0.04	0.013	0.077	0.004	0.11
Mycorrhiza				***	***	**	***	*
Salinity				***	***	***	***	*
Mycorrhiza × Salinity				***	***	***	*	*
Infection				***	***	***	***	*
Infection × Mycorrhiza				***	***	**	***	**
Infection × Salinity				ns	ns	**	***	*
Infection × Salinity × Mycorrhiza				***	*	***	***	*

*^a^Values are the means of three replicates ± SD, where, −AM = non-mycorrhizal, +AM = mycorrhizal, S_0_ = salinity level of 0.7, S_1_ = salinity level of 2.3, S_2_ = salinity level of 3.5 dS m^–1^, −P = non-infected, and +P = infected with *Fusarium* root rot.*

* = significant at p < 0.05, ** = significant at p < 0.01, and *** = significant at p < 0.001.

### Effects on the Photosynthetic Pigments

The mean contents of the photosynthetic pigments in banana leaves in response to the colonization with AMF and/or infection with root rot under different levels of salinity are presented in Table 4. The obtained results indicated that the photosynthetic pigments in banana leaves declined due to the salinity level of the soil. Moreover, infection of banana plantlets with root rot also led to a reduction in the photosynthetic pigments. The lowest contents of Chl *a*, Chl *b*, and carotenoids were recorded for the non-mycorrhizal banana plantlets infected with root rot under salinity level S1 compared with the untreated control plantlets after 4 and 10 weeks api. In contrast, the colonization of banana roots with AMF significantly enhanced these contents in both harvests when compared with the non-mycorrhizal plantlets. The highest contents of the photosynthetic pigments were recorded for the non-mycorrhizal infected banana plantlets under the salinity level S0 after 4 and 10 weeks api. In addition, the root colonization of the infected plantlets under different salinity levels significantly lowered the negative effects resulting from these stresses compared with the non-mycorrhizal stressed plantlets.

**TABLE 4:** Photosynthetic pigments (μg g^–1^ fresh weight) of banana shoots in response to colonization with arbuscular mycorrhizal fungi and/or infection with *Fusarium* root rot under different levels of salinity^*a*^.

Mycorrhizal status	Salinity level	Infection	Weeks after pathogen inoculation					
			4			10		
			Chlorophyll *a*	Chlorophyll *b*	Carotenoids	Chlorophyll *a*	Chlorophyll *b*	Carotenoids
−M	S0	−P	443 ± 2.53	230 ± 2.11	198 ± 1.10	487 ± 2.35	266 ± 1.99	218 ± 2.11
		+P	424 ± 1.51	219 ± 1.51	183 ± 0.95	441 ± 2.41	233 ± 1.74	196 ± 2.03
	S1	−P	436 ± 2.37	224 ± 1.77	175 ± 0.96	465 ± 2.20	241 ± 1.80	181 ± 1.49
		+P	415 ± 3.01	202 ± 1.40	139 ± 1.04	427 ± 1.79	218 ± 1.93	153 ± 1.65
	S2	−P	427 ± 2.66	215 ± 0.97	148 ± 1.21	442 ± 1.91	233 ± 2.03	161 ± 1.30
		+P	420 ± 2.15	204 ± 2.03	141 ± 0.96	431 ± 2.31	224 ± 1.99	150 ± 2.01
+M	S0	−P	486 ± 3.10	259 ± 2.14	210 ± 1.05	512 ± 3.10	284 ± 2.31	244 ± 1.44
		+P	439 ± 2.54	227 ± 1.78	193 ± 0.78	457 ± 2.27	265 ± 1.64	220 ± 1.96
	S1	−P	448 ± 2.78	240 ± 1.32	187 ± 074	481 ± 2.91	272 ± 1.97	208 ± 0.97
		+P	425 ± 1.47	218 ± 1.02	150 ± 1.17	440 ± 3.03	245 ± 2.07	178 ± 0.68
	S2	−P	438 ± 1.85	227 ± 1.05	162 ± 0.86	456 ± 2.69	269 ± 2.31	183 ± 1.01
		+P	430 ± 0.98	221 ± 1.24	158 ± 0.94	449 ± 2.85	251 ± 2.34	175 ± 1.09
LSD 0.05			7.9	6.75	2.31	8.41	9.37	4.08
Mycorrhiza			***	***	***	***	***	***
Salinity			***	***	***	***	***	***
Mycorrhiza × Salinity			***	***	***	***	***	***
Infection			**	**	**	**	**	*
Infection × Mycorrhiza			**	**	**	***	**	**
Infection × Salinity			**	***	**	***	***	**
Infection × Salinity × Mycorrhiza			***	***	**	***	***	***

*^a^Values are the means of three replicates ± SD, where, +AM = mycorrhizal, −AM = non-mycorrhizal, S_0_ = salinity level of 0.7, S_1_ = salinity level of 2.3, S_2_ = salinity level of 3.5 dS m^–1^, −P = non-infected, and +P = infected with *Fusarium* root rot.*

* = significant at p < 0.05, ** = significant at p < 0.01, and *** = significant at p < 0.001.

### Effects on the Disease Incidence and Severity

The disease incidence and severity of banana plantlets in response to the colonization with AMF, and/or infection with root rot under different levels of salinity are presented in Table 5. The results showed that salinity stress increased the DS of the infected banana plantlets compared with the unstressed-infected plantlets, whereas the DI was not affected by the salinity level, recording 100% under the three tested levels. The highest DS was recorded for the infected plantlets under salinity level S1, whether at 4 or 10 weeks api, and the lowest under salinity level S0, with an intermediate DS at salinity level S2. The colonization of the banana roots with AMF reduced both the DS and DI of the infected plantlets under the different salinity levels compared with the non-mycorrhizal plantlets at 4 and 10 weeks api. Among the mycorrhizal-infected plantlets, the banana plantlets infected with root rot under salinity level S1 recorded the highest DS and DI, compared with the other salinity levels.

**TABLE 5:** Disease severity and incidence of banana plants in response to colonization with arbuscular mycorrhizal fungi and/or infection with *Fusarium* root rot under different levels of salinity^*a*^.

Mycorrhizal status	Salinity level	Infection	Weeks after pathogen inoculation			
			4		10	
			Disease incidence (%)	Disease severity (%)^*b*^	Disease incidence (%)	Disease severity (%)
−M	S0	−P	0	0	0	0
		+P	100 ^a^	40 ^c^	100 ^a^	66.7 ^c^
	S1	−P	0	0	0	0
		+P	100 ^a^	53.3 ^a^	100 ^a^	80 ^a^
	S2	−P	0	0	0	0
		+P	100 ^a^	46.7 ^b^	100 ^a^	73.3 ^b^
+M	S0	−P	0	0	0	0
		+P	73.3 ^c^	33.3 ^d^	75 ^c^	46.7 ^e^
	S1	−P	0	0	0	0
		+P	80 ^b^	46.7 ^b^	100 ^a^	66.7 ^c^
	S2	−P	0	0	0	0
		+P	73.3 ^c^	40 ^c^	80 ^b^	53.3 ^d^

*^a^Values are the means of three replicates ± SD, where, +AM = mycorrhizal, −AM = non-mycorrhizal, S_0_ = salinity level of 0.7, S_1_ = salinity level of 2.3, S_2_ = salinity level of 3.5 dS m^–1^, −P = non-infected, and +P = infected with *Fusarium* root rot.*

*^b^Disease severity (DS) was estimated according to Carling et al. (1999).*

### Effects on the Mycorrhizal Colonization

**FIGURE 3:**
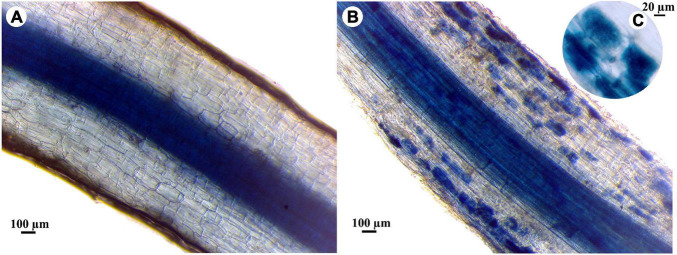
Light micrographs showing the mycorrhizal colonization in banana roots, where, **(A)** non-mycorrhizal root, **(B)** mycorrhizal root, and **(C)** highly magnified image of colonized root showing arbuscules.

The mycorrhizal colonization percentages of banana roots in response to the infection with root rot and/or different levels of salinity are presented in Table 6. No mycorrhizal colonization was detected in the banana plantlets without the AMF inoculum. In contrast, all the plantlets treated with the AMF showed varied levels of mycorrhizal colonization. The mycorrhizal colonization in the banana roots was examined using a light microscope showing typical mycorrhizal structures (Figure 3). The data obtained showed that the mycorrhizal colonization in banana plantlets was inversely proportional to the salinity level. The highest colonization levels were recorded for the uninfected-mycorrhizal banana plantlets under salinity level S0. The infection of banana plantlets with root rot reduced the mycorrhizal colonization in their roots under all tested salinity levels, at 4 and 10 weeks api, compared with the uninfected-mycorrhizal plantlets.

**TABLE 6:** Mycorrhizal colonization of banana roots in response to infection with *Fusarium* root rot and/or different levels of salinity^*a*^.

Mycorrhizal status	Salinity level	Infection	Weeks after pathogen inoculation					
			4			10		
			F (%)	I (%)	A (%)	F (%)	I (%)	A (%)
−M	S0	−P	0	0	0	0	0	0
		+P	0	0	0	0	0	0
	S1	−P	0	0	0	0	0	0
		+P	0	0	0	0	0	0
	S2	−P	0	0	0	0	0	0
		+P	0	0	0	0	0	0
+M	S0	−P	90.5 ± 1.0	51.6 ± 0.5	23.3 ± 0.7	96.9 ± 1.0	67.3 ± 0.6	28.1 ± 1.0
		+P	84.3 ± 06	44.3 ± 0.4	19.8 ± 0.8	89.1 ± 0.3	53.1 ± 0.8	20.2 ± 0.9
	S1	−P	87.4 ± 1.1	48.4 ± 0.8	19.1 ± 0.6	93.1 ± 0.7	61.1 ± 1.0	23.3 ± 0.5
		+P	79.2 ± 0.8	39.1 ± 1.1	16.5 ± 0.3	83.3 ± 03	49.6 ± 0.7	17.7 ± 0.7
	S2	−P	80.5 ± 1.3	40.1 ± 1.0	15.8 ± 0.9	89.1 ± 0.7	51.3 ± 1.1	19.5 ± 0.8
		+P	68.3 ± 0.9	33.7 ± 0.8	10.3 ± 0.4	71.6 ± 1.1	44.3 ± 0.9	14.3 ± 1.0
LSD 0.05			3.25	3.95	2.63	3.41	4.14	3.08
Mycorrhiza			***	***	***	***	***	***
Salinity			***	**	*	***	***	**
Mycorrhiza × Salinity			***	***	**	***	***	***
Infection			***	***	**	***	***	***
Infection × Mycorrhiza			***	**	**	***	**	**
Infection × Salinity			***	***	***	***	***	***
Infection × Salinity × Mycorrhiza			***	***	**	***	***	**

*^a^Values are the means of three replicates ± SD, where, +AM = mycorrhizal, −AM = non-mycorrhizal, S_0_ = salinity level of 0.7, S_1_ = salinity level of 2.3, S_2_ = salinity level of 3.5 dS m^–1^, −P = non-infected, +P = infected with Fusarium root rot, F = frequency of colonization, I = intensity of colonization, and A = frequency of arbuscules.*

* = significant at p < 0.05, ** = significant at p < 0.01, and *** = significant at p < 0.001.

## Discussion

**FIGURE 4:**
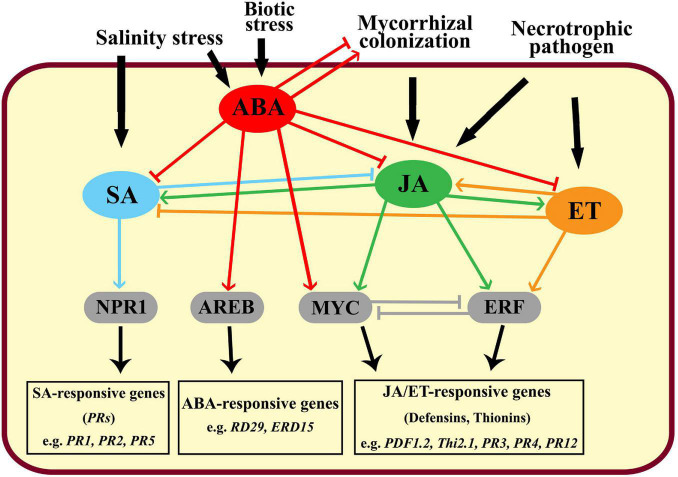
Graphical diagram showing the crosstalks between the different signaling pathways in the plant in response to biotic and abiotic agents, where, SA: salicylic acid, ABA: abscisic acid, JA: jasmonic acid, ET: ethylene, NPR1: non-expressor of pathogenesis-related genes 1, AREB: ABA-response element binding protein, and ERF: ethylene response factor.

Plant responses to different biotic and abiotic stresses have been widely studied (Isayenkov and Maathuis, 2019). In this regard, various stress-responsive mechanisms have been discussed including the induction of signaling molecules, transcription factors, and/or activation of kinase cascades (Ben Rejeb et al., 2014). However, the responsive behaviors of a plant in case of the combined stresses are unique, more complicated, and differ compared with the individual stresses (Suzuki et al., 2014). In this work, effects of the mycorrhizal colonization of banana roots and/or infection with root rot on the transcriptional expression of some stress-responsive genes, selected as marker genes for different signaling pathways, were studied under different levels of salinity. The data obtained in our study revealed that the transcript level of *JERF3* increased in response to the increase in the salinity level. *JERF3* plays a vital role as a transcription activator in the JA and ET-signaling pathways regulating multiple stress-responsive genes through binding to the GCC box in their promoters (Pegoraro et al., 2013). The overexpression of these genes leads to activation and improvement of plant tolerance and resistance to multiple abiotic and biotic stresses (Ku et al., 2018). For example, Zhang et al. (2010) found that the overexpression of *JERF3* in transgenic rice plants improved their tolerance to salinity and drought stresses and significantly increased their soluble sugars and proline contents by triggering some stress-responsive genes, compared with the non-transgenic plants. Our results indicated that *JERF3* was also upregulated by the infection of the banana plantlets with root rot, or mycorrhizal colonization under salinity levels S0 and S2, but not S1. This result is in agreement with those obtained by Zhang et al. (2007) who reported the overexpression of *JERF3* in wheat plants after being infected with *Blumeria graminis*, *F. graminearum*, or *Rhizoctonia cerealis*. *JERF3* was overexpressed by the individual stresses (salinity and infection) but under the combined stresses, the response was different. Under the combined stresses, the plant responses are unpredicted and may differ from those produced under individual stresses because of the interactions and crosstalks between the different signaling pathways they induce, which may be synergistic, antagonistic, or additive. However, one of the most limiting factors affecting plant responses to combined stresses is the intensity of the stress (Ben Rejeb et al., 2014). In our study, the first and superior effect when we combined the abiotic and biotic stresses is exclusively related to the abiotic stress (salinity), because the biotic stimulator (fungal infection or mycorrhizal colonization) requires more time to be established in the plant tissue resulting in delayed plant responses (Sah et al., 2016). Under the abiotic stress, the rapid accumulation of ABA, which is the primary phytohormone concerned with the perception of various abiotic stresses, modulates a set of plant responses via the ABA-responsive element-binding protein (AREB)/ ABA-responsive element-binding factor (ABF) transcription factors activating the plant tolerance (Sah et al., 2016). The mechanisms involved include inducing stomatal closure, production of osmoprotectants, and accumulation of nitric oxide (Hewage et al., 2020). In addition, plants synthesize and accumulate ABA in response to biotic stresses such as pathogen attacks which leads to triggering the plant immunity (Bharath et al., 2021). In this concern, the accumulation of ABA may induce stomatal closure to prevent the pathogen entry, elicit defense responses, and/or trigger callose deposition to act as a physical barrier limiting the pathogen attack and spread (Hewage et al., 2020). Biotic stress resistance is also mediated by the phytohormones SA (via the key regulator *NPR1*), JA (*via* the transcription factors *MYC*/*ERF*), and/or ET (via the ethylene-responsive factor *ERF*) (Ku et al., 2018; Backer et al., 2019). However, the role of ABA in plant resistance is more complicated for biotic stresses than for abiotic stresses. The involvement of ABA in the plant immunity against pathogen attacks may often be interlinked with the SA, JA, and ET signaling pathways, which are more dedicated to the plant defense against biotic stresses than ABA (Hewage et al., 2020). Under the combined abiotic and biotic stresses, ABA mostly antagonizes SA, JA, and ET actions elevating plant susceptibility to the pathogen, particularly at the early stages where they are not yet be induced (Nahar et al., 2012). In contrast, ET synergistically regulates the JA-signaling pathway *via* the *ERF*-branch upon the attack of necrotrophic pathogens (Li N. et al., 2019). Moreover, ABA is biosynthesized by the fungal pathogen itself as an effector molecule (Lievens et al., 2017), owing to its inducing effect on the virulence and pathogenicity capabilities of the pathogen such as appressoria formation and spore germination, in addition to their role in hindering the host resistance (Spence et al., 2015). Yazawa et al. (2012) found that minimizing the ABA levels in rice plants increased their resistance to *Magnaporthe oryzae*, the causing agent of rice blast, by impairing the pathogen penetration ability and leading to a reduction in the disease incidence. However, their inducing effect on the plant defense was also reported (Vos et al., 2019). Furthermore, ABA modulates the mycorrhizal establishment by inducing the AMF colonization at low levels but impairs it at the high ABA levels (Charpentier et al., 2014). These opposite roles make ABA a multifaceted molecule that switches from a synergistic to an antagonistic effect, and as a control point between abiotic and biotic stress (Ton et al., 2009). The probable crosstalks between different signaling pathways under the combined stresses are illustrated in Figure 4.

Mycorrhizal colonization, which is one of the key factors in the present study, is widely known to trigger JA-dependent plant defense responses to different pathogens (Aseel et al., 2019; Rashad et al., 2020a) and induce plant tolerance to salinity and drought (Evelin et al., 2019). The results obtained in our study showed that the overexpression of *JERF3* due to mycorrhizal colonization and/or fungal infection depended on salinity level. In other words, the limiting effect of the salinity level had superiority over the other stresses during the experiment. The overexpression of *JERF3* obtained by the treatments applied upregulated the expression of other stress-responsive genes enhancing plant tolerance against abiotic and biotic stresses indicating their important role as transcriptional regulators in banana plantlets. However, the observation differed under combined stresses compared with individual stresses. The interconnections between the different signaling pathways (ABA, SA, JA, and ET) may regulate *JERF3* expression. Mycorrhizal colonization and necrotrophic infection triggered the JA and ET signaling pathway through the overexpression of *JERF3*, while the salinity stress mainly induced ABA signaling, which antagonized the JA and ET pathways, and downregulated the *JERF3* expression. The overexpression of *JERF3* obtained in this study is in accordance with the one reported by Velivelli et al. (2015) in mycorrhizal potato plantlets.

In this study, the same inducing behaviors of the applied treatments were also recorded for the *POD*, *PR1*, *CHI*, and *GLU* genes. The *POD* encoding gene is a plant-specific oxidoreductase, which is involved in many physiological processes such as lignin polymerization, plant development, resistance to pathogens attack, and tolerance to abiotic stresses. Their antioxidant activity is exerted by oxidizing the phenolic compounds regulating the ROS and free radicals produced due to abiotic and biotic stresses (Shigeto and Tsutsumi, 2016). The induction of *POD* in response to salinity stress, fungal infection, or mycorrhizal colonization has been previously reported (El-Sharkawy et al., 2018; Cai and Gao, 2020; Rashad et al., 2020a). Moreover, the modulation of AMF symbiosis to the antioxidant responses in the salinity-stressed plants was also reported by Evelin and Kapoor (2014). The overexpression of *JERF3* leads to the induction of many pathogenesis-related (PR) genes enhancing the plant resistance to many biotic and abiotic stresses (Ku et al., 2018). This result is in agreement with that obtained in this study regarding the overexpression of the *PR1*, *CHI*, and *GLU* genes. The *PR1* gene encodes a protein with antifungal activity and is involved in plant resistance against many fungal pathogens *via* the SA-signaling pathway (Breen et al., 2017). The *CHI* gene (*PR3*) encodes the chitinase enzyme, which is involved in plant defense against different fungal infections *via* the JA-signaling pathway. The *CHI* gene catalyzes the degradation of the chitin component in the fungal cell walls by hydrolyzing the β-1,4 bonds between their subunits (Zheng et al., 2020). The *GLU* gene (*PR2*) encodes the antifungal enzyme β-1,3-glucanase, which is involved in the plant resistance to pathogen attack *via* the SA-signaling pathway, by catalyzing the hydrolysis of the β−1,3−glycosidic bond in the 1,3-glucan molecules in fungal cell walls (Gupta et al., 2013). Moreover, the induction of *CHI* and *GLU* under salinity stress has been also reported (Ohnuma et al., 2011; Su et al., 2016). In this regard, most likely, the mechanisms contributing to salt stress tolerance seem to be the inhibition of the Na^+^ influx to minimize their toxicity, modulation of the cell wall metabolism to regulate root cross-linking, nutrient translocation, and osmotic changes, as well as releasing ABA from ABA-glucosyl esters (Byrt et al., 2018; Liu et al., 2019).

Our results indicated the triggering effect of AMF symbiosis on the defense responses of banana plantlets against root rot infection, even under salt-stress conditions. However, the salinity level showed a limiting effect under combined stresses, even in mycorrhizal plantlets. The biocontrol effect of mycorrhizal colonization on plant resistance against different pathogens was investigated in diverse sets of crops and pathogens (El-Sharkawy et al., 2018; Aseel et al., 2019). Once the mycorrhizal symbiosis was established in the plant root, a wide-ranging genetic reprogramming proceeded in the plant, resulting in an array of metabolic modulations and leading to an induction of both innate and adaptive plant immunity (Vangelisti et al., 2018). However, the modulation intensity depends on various factors including the arbuscular mycorrhizal fungus, the plant species, the developmental phase of symbiosis, and the surrounding environmental conditions (Jung et al., 2012). Various defense mechanisms have been discussed in this concern including cell wall lignification, the accumulation of polyphenolic compounds and defensive proteins, and the activation of antioxidant and pathogenesis-related enzymes (Abdel-Fattah et al., 2011; Rashad et al., 2020a). In addition, the growth-promoting and nutrient uptake enhancing effects to ameliorate the adverse influences of the infection have also been reported (Campo et al., 2020; Rashad et al., 2020b). On the other hand, the mycorrhizal colonization of the banana plantlets alleviated the adverse effects due to salinity stress. The different mechanisms used by AMF to trigger the salinity tolerance in plants have been discussed, including the production of osmoregulators such as proline, accumulation of antioxidant molecules, and induction of antioxidant enzymes, improvement of water uptake efficiency, and improvement of stomatal conductance (Dastogeer et al., 2020). However, the stress-mitigating effects of AMF depend on many factors such as type of the host plant, species of mycorrhizal fungus, duration and stage of colonization, environmental conditions, and stresses level, and complex interactions. In our study, mycorrhizal colonization alleviated the adverse effects of salinity and infection to some extent depending on the salinity level and its combination with the infection stress. Our results showed the enhancing effect of mycorrhizal colonization on the growth of banana plantlets under different salinity levels. The growth-promoting effect of AMF has been extensively reported in various crops (El-Sharkawy et al., 2018; Rashad et al., 2020a). The inducement of various plant growth regulators such as auxins, cytokinins, and gibberellins during root mycorrhizal colonization has been reported (Martín-Rodríguez et al., 2016). In addition, the enhancement of plant photosynthesis, cell metabolism, and water and nutrient acquisition by AMF symbiosis has been also reported (Iqbal et al., 2021), which is in agreement with our results on the enhancement of the photosynthetic pigments and nutrient contents in banana plantlets by mycorrhizal colonization.

The nutrient content of the plant affects, positively or negatively, its immunity against invading pathogens. Different physiological functions for the plant nutrients have been discussed. For example, silicon has an inducing effect on cell wall lignification and deposition of antimicrobial phenolic compounds and phytoalexins at the infection sites. These defensive mechanisms limit the fungal spread and inhibit its ability to form haustoria (Van Bockhaven et al., 2013). Calcium has a key role in pathogen recognition, cell wall stability, and inhibition of polygalacturonase enzyme. This enzyme is secreted by the pathogen as a virulence factor during the penetration stage (Dordas, 2008). In our study, the effects of different studied stresses on the contents of the three main essential nutrients in plants (N, P, and K) were investigated. In addition, the contents of Na and Cl, the main constituent ions in saline soil, were also studied. Nitrogen is one of the most important essential nutrients in plants, being the main constituent of many vital structural and functional compounds in plants such as nucleic acids, proteins, and enzymes. Moreover, it is considered the limiting nutrient for plant growth and productivity (Liu et al., 2018). Phosphorus is a major essential nutrient for plant growth. It has multiple vital functions in plants including energy transfer, photosynthetic efficiency, nutrient transfer, and genetic traits transfer to the next generations (Malhotra et al., 2018). Potassium is one of the most important essential nutrients and has many functional roles in plant metabolism and development. It acts as an enzyme activator in various physiological functions including protein synthesis, N metabolism, glucose transportation, and cell elongation. Moreover, it has vital roles in the regulation of stomatal conductance, cellular osmotic pressure, and electrolyte balance (Hu et al., 2016). In contrast, despite the fact that Na and Cl are considered micronutrients for plants, they have toxic effects on plant growth at their high concentrations. Under salinity conditions, they reduce soil water uptake, increase osmotic stress, reduce cellular ion imbalance, cause ion toxicity, and inhibit enzymatic activities and cellular functions (Isayenkov and Maathuis, 2019). In addition, oxidative stress can also occur due to the production of ROS that attacks plant tissues and DNA (Kamran et al., 2020). In general, any disturbance in the plant nutrient content, whether deficiency or oversupply, may affect plant development, productivity, stress tolerance, and immunity. Our results indicated the decline in the N, P, K contents in non-mycorrhizal banana plantlets due to infection with *F. solani*. During the infection process, the nutrition acquisition by the pathogen plays a vital role in establishing a successful infection and effective spreading. Once the necrotrophic fungus penetrates the plant cell, it produces toxins and degrading enzymes causing the lysis of the host cell. This makes the cell nutrients available to the pathogen by direct absorption or transporters (Divon and Fluhr, 2007). In addition, the adverse effects due to the infection extend to include the destruction of the root morphology and physiology leading to a reduction in the water and nutrients uptake from soil. Our results showed that the N, P, K contents in banana leaves were adversely affected under salinity conditions due to the reduced soil nutrient availability, plant uptake efficiency, and nutrients translocation inside the plant. Whereas, the Na and Cl contents increased in the banana leaves with the increase in soil salinity. The imbalance in the nutrient contents and disturbance in the osmotic potential due to salinity stress led to growth retardation in the banana plantlets. In contrast, the mycorrhizal colonization of the banana plantlets enhanced the N, P, K contents in their leaves. This is due to the enhancing effect of AMF on nutrients uptake *via* their extraradical mycelium network, which explores larger soil areas. Moreover, AMF produces organic acids and enzymes such as phosphatases in the soil, which facilitate the nutrients released from the soil, and enhance their availability to the plant (Saia et al., 2014; Iqbal et al., 2021). Furthermore, the mycorrhizal colonization of the banana plantlets alleviated the adverse effects due to the infection and/or salinity stresses, compensated for the nutrient deficiency due to both stresses, and maintained the ionic homeostasis, which led to the enhancing effect on the banana growth. The enhancement of the plant nutrient uptake by the mycorrhizal colonization makes the plant more vigorous, and more resistant to stresses. In addition, as we mentioned before, the mycorrhizal colonization of the banana plantlets induced plant resistance against the infection with root rot and salinity stress *via* multiple defensive mechanisms. These mechanisms reduced the adverse effects due to the applied stresses.

Our results showed that the infection of the banana plantlets with necrotrophic pathogens, and/or exposure to salinity stress led to a reduction in their photosynthetic pigments content. This may be attributed to the reduction in water uptake and transport due to root damage. The water deficiency induces stomatal closure to minimize the water loss, which leads to a reduction in the carbon dioxide uptake and the photosynthesis process (Walters, 2015). Carbon starvation is another proposed mechanism during necrotrophic fungal infection. Upon infection, downregulation of the genes encoding carbon and starch metabolism occurs, which leads to a reduction in carbon assimilation and transport (Li P. et al., 2019). The mycorrhizal colonization of banana plantlets alleviates the adverse effects due to the infection and salt stresses on photosynthesis. The mechanisms used by AMF to alleviate these detrimental effects most likely include the improvement of the photosynthesis and gas exchange capacities, enhancement of the content of the photosynthetic pigment, amelioration of water and nutrient absorption and use efficiency, especially phosphorous, improvement in Na^+^ exclusion capacity, and induction of antioxidant activities (Romero-Munar et al., 2019). Triggering salinity tolerance in different plants by AMF colonization has been widely studied (Evelin et al., 2019; Li et al., 2020).

One of the adverse effects of salinity stress on plants is the alteration of their root morphology. In this regard, the thinning, elongation, and discoloration of banana roots were observed under salt stress. This result is consistent with that of Arif et al. (2019) who reported up to 91% increase in the length and density of root hairs of rapeseed plants under salt stress. The banana plantlets tended to adapt to the salinity stress to decrease its negative effects. Root elongation under saline conditions is an adaptive mechanism to increase the nutrient uptake ability. On the other hand, the infection of the banana plantlets with *F. solani* led to morphological alterations in the infected roots causing necrotic lesions, pigmentation, thinning, and weakness of the root, which affect the plant growth. In contrast, mycorrhizal colonization induced some morphological alterations as adaptive mechanisms in banana roots under salinity stress and/or infection. These alterations include an increase in the root volume and lateral branching to maximize the absorptive surface area and hence uptake more water and nutrients to compensate for their deficiency due to salt stress. The thickening of the root system is another adaptive morphological alteration to decrease both Na^+^ influxes into the root tissue as well as the outflow of water and nutrients outside the root tissue (Sheng et al., 2009). In addition, root thickening forms a physical barrier against infection penetration.

In conclusion, the present study indicated that the mycorrhizal colonization of the banana plantlets triggered, at varying degrees, the transcriptional expression of the stress-responsive genes *JERF3*, *POD*, *PR1*, *CHI*, and *GLU*, which mediate different signaling pathways, mainly JA. However, their inducing effect was significantly affected by the salinity level at which the symbiosis and/or infection occurred. In addition, the mycorrhizal colonization of the banana plantlets improved their growth, photosynthesis, and nutrient uptake. Moreover, the mycorrhizal colonization greatly alleviated the detrimental effects of salt and infection stresses on banana plantlets despite their negative influences on the colonization level. In general, our results indicated that the responses of banana plantlets under combined stresses are differed from those under individual stresses, and are differentially affected by the level of salinity stress.

## Data Availability Statement

The datasets presented in this study can be found in online repositories. The names of the repository/repositories and accession number(s) can be found in the article/supplementary material.

## Author Contributions

YR conceived the idea and the design of the work, contributed to the implementation of the greenhouse experiment, mycorrhizal estimation, molecular investigation, and helped in writing the manuscript and photo editing. WF contributed to the design of the work, greenhouse experiments, and nutrient analyses. MS contributed to the photosynthesis determination and molecular investigation. NE contributed to the greenhouse experiment and statistical analyses. All authors revised and approved the final manuscript.

## Conflict of Interest

The authors declare that the research was conducted in the absence of any commercial or financial relationships that could be construed as a potential conflict of interest.

## Publisher’s Note

All claims expressed in this article are solely those of the authors and do not necessarily represent those of their affiliated organizations, or those of the publisher, the editors and the reviewers. Any product that may be evaluated in this article, or claim that may be made by its manufacturer, is not guaranteed or endorsed by the publisher.
